# Supplementary material for: Restricted MHC-II trafficking in Mycobacterium tuberculosis-infected M2-like macrophages limits CD4+ T cell activation
Source: bioRxiv. 2026 Jul 10:2026.07.07.736943. Preprint. [Version 1] doi: 10.64898/2026.07.07.736943 (PMC13370412; doi:10.64898/2026.07.07.736943)
Supplement: 1 [file NIHPP2026.07.07.736943v1-supplement-1.pdf]

bioRxiv preprint doi: https://doi.org/10.64898/2026.07.07.736943; this version posted July 10, 2026. The copyright holder for this preprint (which was not certified by peer review) is the author/funder, who has granted bioRxiv a license to display the preprint in perpetuity. It is made available under a CC-BY 4.0 International license.

Supplemental Figures

Supplemental Figure 1 (related to Figure 3). M1-like Mtb-infected macrophages show decreased expression of *HERC5* and *IL10RA*. (A) Venn diagram depicting DEGs between non-infected M2-like macrophages and after Mtb infection (light gray) compared to DEGs between non-infected M1 and M2-like macrophages (blue). **(B)** Significant (adjusted) pathways (Reactome) from 1061 genes upregulated by M2-like macrophages after Mtb infection. Pathway enrichment significance was determined by Fisher's exact test with Benjamini-Hochberg correction for multiple comparisons, as implemented in Enrichr. **(C)** Venn diagrams depicting DEGs between non-infected M1-like macrophages and after Mtb infection (orange) and DEGs between non-infected M1 and M2-like macrophages (blue). **(D)** Venn diagrams depicting DEGs unique to Mtb-infected M1-like macrophages (orange) after removing upregulated DEGs in common with M1 and M2-like macrophages after Irrad. Mtb (compared to non-infected, grey). **(E)** Bar graph showing mean (+/- SD) IFN-β concentrations in supernatants of infected M1 and M2-like macrophages 24 h post infection, in triplicate, representative of 3 independent experiments (3 individuals). **(F) (Left)** Representative histogram and **(right)** bar graph showing MFI (mean + SD) of IFNAR1, in triplicate, in M1 and M2-like MDMs, representative of 2 independent experiments (3 individuals). **(G)** Bar graphs showing the mean mRNA concentrations (+/- SD) of *RSAD2* and *HERC5* and **(H)** *IL10RA* in non-infected and infected M2 and M1-like MDMs, in duplicate, representative of 2 independent experiments (4 individuals). Significance was

bioRxiv preprint doi: https://doi.org/10.64898/2026.07.07.736943; this version posted July 10, 2026. The copyright holder for this preprint (which was not certified by peer review) is the author/funder, who has granted bioRxiv a license to display the preprint in perpetuity. It is made available under a CC-BY 4.0 International license.

determined using either Student's t-test or one-way ANOVA with Sidak's post-test corrected for multiple comparisons. ** $p < 0.01$; ****$p < 0.0001$; ns, not significant.

Supplemental Figure 2 (related to Figure 4). MHC-I trafficking is not restricted in Mtb-infected M2-like macrophages. (A) Fluorescence microscopy of M1 and M2-like MDMs treated with anti-HLA-DR mAb blockade (clone L243), infected with Mtb-GFP showing surface anti-HLA-DR staining (BV421-LN3) and re-stained with HLA-DR (PE-L243) 24 h post infection. Images taken at 20x magnification. **(B)** Fluorescence microscopy of M1 and M2-like MDMs treated with anti-HLA-ABC mAb blockade (clone W6/32), infected with Mtb-GFP showing surface anti-HLA-ABC staining (BV421-G46-2.6) and re-stained with HLA-ABC (PE-W6/32) 24 h post infection. Images taken at 20x magnification. **(C) (left)** Bar graph of the percentage (mean ± SD) of infected macrophages showing positive HLA-ABC (PE-W6/32) re-staining, representative of 3 independent experiments (3 individuals) and **(right)** Bar graph showing MFI (mean + SD) of post-infection re-stained HLA-ABC (PE-W6/32) in triplicate, representative of 3 independent experiments (3 individuals). **(D) (left)** Representative histogram and **(right)** bar graph showing MFI (mean + SD) of post-infection HLA-DR-bound surface CLIP in triplicate, representative of 4 independent experiments (2 individuals). **(E) (left)** Representative histogram and **(right)** bar graph showing MFI (mean + SD) of post-infection total CD74 in triplicate, representative of 3 independent experiments (3 individuals). **(F) (left)** Representative histogram and **(right)** bar graph showing MFI (mean + SD) of post-infection total HLA-DM in triplicate, representative of 3 independent experiments (3 individuals). Significance was determined using Student's t-test. ** $p < 0.01$; ****$p < 0.0001$; ns, not significant.

bioRxiv preprint doi: https://doi.org/10.64898/2026.07.07.736943; this version posted July 10, 2026. The copyright holder for this preprint (which was not certified by peer review) is the author/funder, who has granted bioRxiv a license to display the preprint in perpetuity. It is made available under a CC-BY 4.0 International license.

Supplemental Figure 3 (related to Figure 5). *HERC5* knockdown does not alter infectivity or viability of M2-like macrophages. (**A)** Bar graph showing mean (+ SD) mRNA concentrations of *B2M* **(left)** and *HERC5* **(right)** in infected M2-like macrophages receiving either non-targeting (NT) siRNA or *HERC5* siRNA in triplicate, representative of 3 independent experiments (3 individuals). **(B)** Bar graphs showing mean (+ SD) **(left)** percentage of Live/Dead low M1 and M2-like macrophages under each indicated condition, **(middle)** percentage of Mtb-GFP infected M1 and M2-like macrophages under each indicated condition, and **(right)** MFI of Mtb-GFP in infected M1 and M2-like macrophages under each indicated condition in duplicate, representative of 3 independent experiments (3 individuals). **(C)** Bar graph showing MFI (mean + SD) of post-infection **(left)** surface HLA-DR and **(right)** CD274 in M1 and M2-like macrophages under each indicated condition in duplicate, representative of 3 independent experiments (3 individuals). **(D)** Bar graph showing mean (+ SD) mRNA concentrations of *B2M* **(left)** and *HERC5* **(right)** in infected M1, M2-like macrophages, and infected M2-like macrophages receiving IL-10 neutralization in duplicate, representative of 3 independent experiments (3 individuals). **(E)** Fluorescence microscopy of M2-like MDMs under indicated conditions treated with anti-HLA-DR mAb blockade (clone L243), infected with Mtb-GFP showing surface anti-HLA-DR staining (BV421-LN3) and re-stained with HLA-DR (PE-L243) 24 h post infection. Images taken at 20x magnification. Significance was determined using either Student's t-test **(A)** or one-way ANOVA with Sidak's post-test corrected for multiple comparisons **(B, C,** and **D)**. ** $p < 0.01$; ****$p < 0.0001$; ns, not significant.

Extended Data 1 (Related to Figures 2 and 3) includes lists of differentially expressed genes (DEGs) between M1- and M2-like MDMs, and BAL macrophages in pairwise comparisons, as indicated in Figures 2 and 3. **Extended Data 2 (Related to Figures 2 and 3)** includes the gene

bioRxiv preprint doi: https://doi.org/10.64898/2026.07.07.736943; this version posted July 10, 2026. The copyright holder for this preprint (which was not certified by peer review) is the author/funder, who has granted bioRxiv a license to display the preprint in perpetuity. It is made available under a CC-BY 4.0 International license.

lists used to construct the Venn diagrams capturing the DEGs in M1- and M2-like MDMs, and BAL macrophages in Figures 2 and 3.

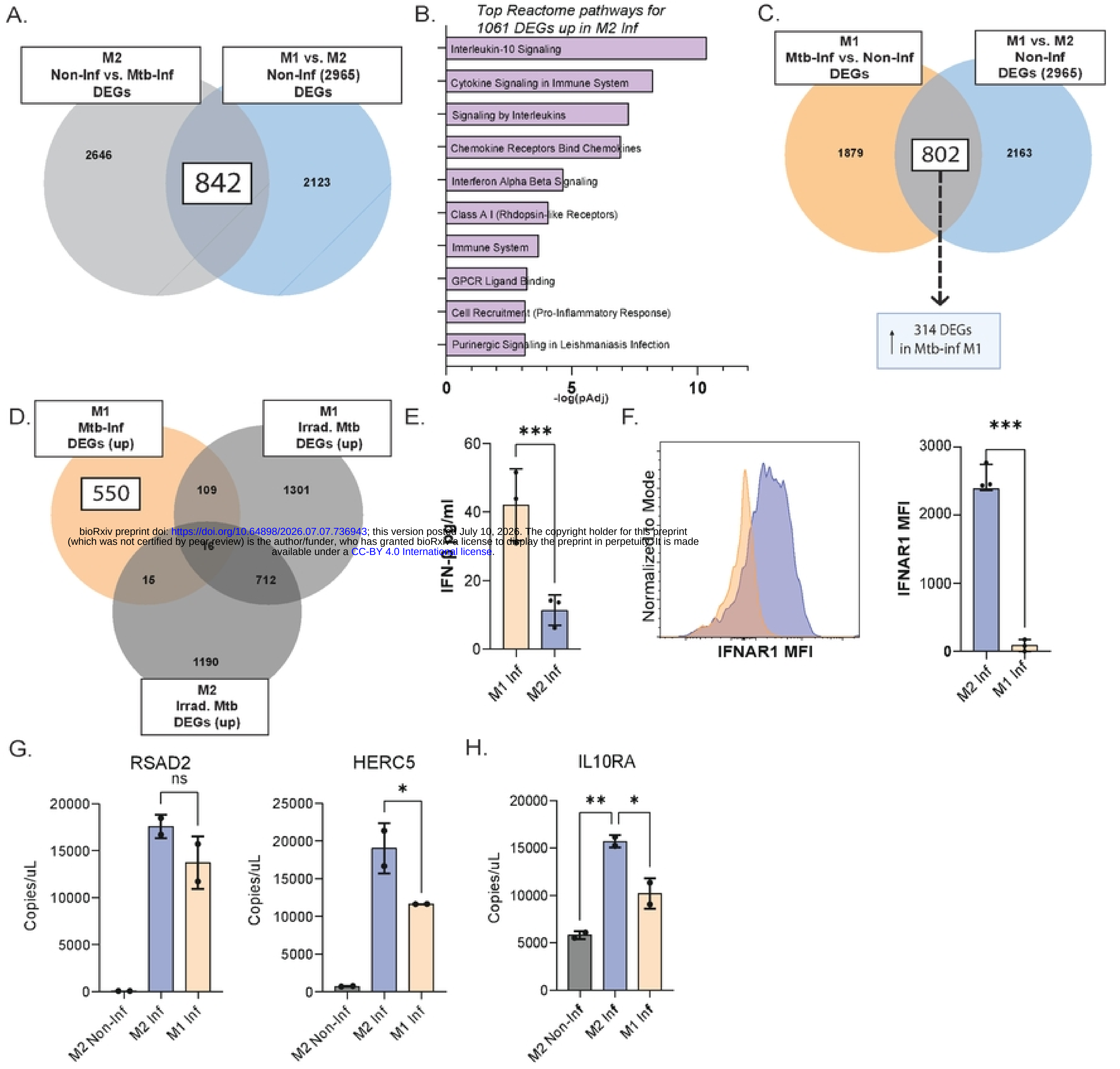

bioRxiv preprint doi: https://doi.org/10.64898/2026.07.07.736943; this version posted July 10, 2026. The copyright holder for this preprint (which was not certified by peer review) is the author/funder, who has granted bioRxiv a license to display the preprint in perpetuity. It is made available under a CC-BY 4.0 International license.

Supplemental Figure 1

Supplemental Figure 1

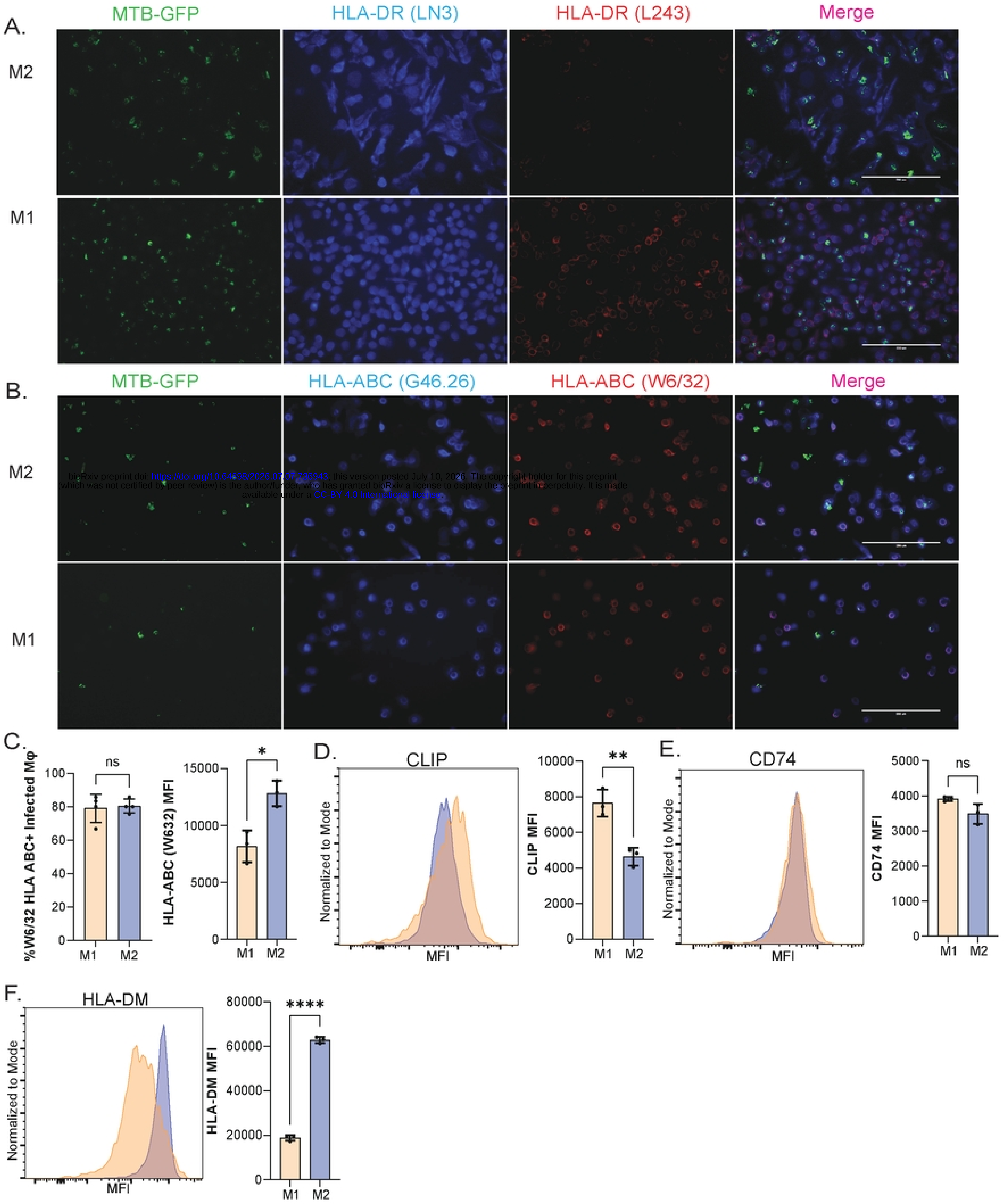

bioRxiv preprint doi: https://doi.org/10.64898/2026.07.07.736943; this version posted July 10, 2026. The copyright holder for this preprint (which was not certified by peer review) is the author/funder, who has granted bioRxiv a license to display the preprint in perpetuity. It is made available under a CC-BY 4.0 International license.

Supplemental Figure 2

Supplemental Figure 2

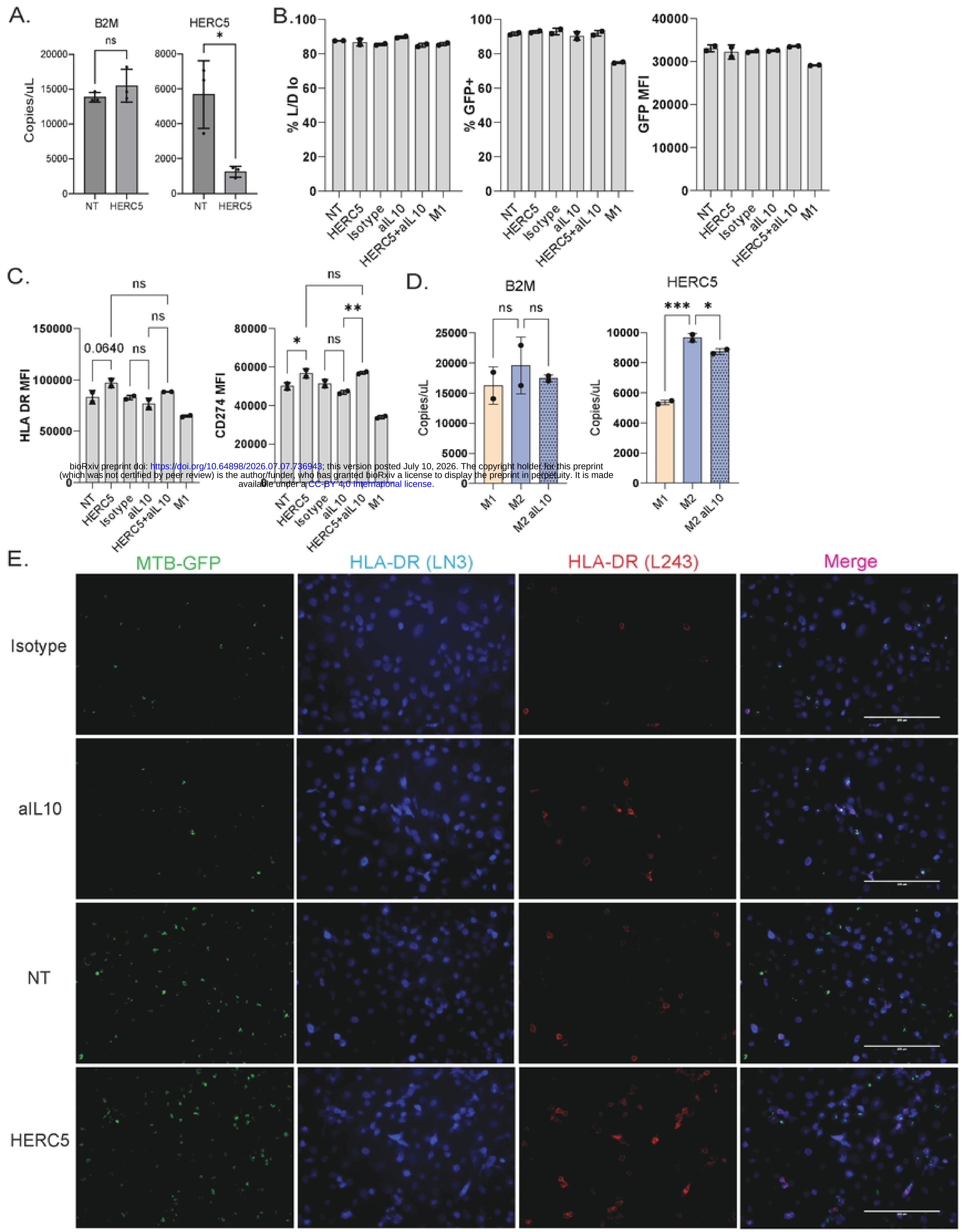

bioRxiv preprint doi: https://doi.org/10.64898/2026.07.07.736943; this version posted July 10, 2026. The copyright holder for this preprint (which was not certified by peer review) is the author/funder, who has granted bioRxiv a license to display the preprint in perpetuity. It is made available under a CC-BY 4.0 International license.

Supplemental Figure 3

Supplemental Figure 3
